# Cervical precancerous changes and selected cervical microbial infections, Kiambu County, Kenya, 2014: a cross sectional study

**DOI:** 10.1186/s12879-017-2747-4

**Published:** 2017-09-25

**Authors:** Evalyne Wambui Kanyina, Lucy Kamau, Margaret Muturi

**Affiliations:** 1 Ministry of Health, P. O. Box 30016-00100, Nairobi, Kenya; 2 Department of Zoological Sciences, Kenyatta University, School of Pure and Applied Sciences, P. O. Box 43844-00100, Nairobi, Kenya; 3 Department of Medical Laboratory Sciences, Kenyatta University, School of Medicine, P. O. Box 43844-00100, Nairobi, Kenya

**Keywords:** Cervical precancerous changes, Cervical intraepithelial neoplasia (CIN), Cervical microbial infections, Pap smear, Kenya

## Abstract

**Background:**

Cervical cancer is the predominant cancer among women in Kenya and second most common in women in developing regions. Population-based cytological screening and early treatment reduces morbidity and mortality associated with the cancer. We determined the occurrence of cervical precancerous changes and cervical microbial infections (*Trichomonas vaginalis, Candida albicans, Neisseria gonorrhea and Actinomyces*) among women attending Family Health Option Kenya (FHOK) clinic in Thika.

**Methods:**

This was a hospital based cross sectional study among women attending reproductive health screening clinic from November 2013 to January 2014. Cervical Intraepithelial Neoplasia (CIN) I, II, III, cervical cancer and microbial infection (*Actinomyces, Trichomonas vaginalis* and Yeast cells) diagnosis was based on Pap smear screening test and High Vaginal Swab wet preparation microscopy. *Neisseria gonorrhea* was diagnosed through Gram staining. Socio-demographic and reproductive health data was collected using a structured questionnaire administered to the study participants and analyzed using Epi Info version 3.5.1.

**Results:**

Of the 244 women screened, 238 (97.5%) presented with cervical inflammation, 80 (32.8%) cervical microbial infections and 12 (4.9%) cervical precancerous changes; 10 (83.3%) with CIN I and 2 (16.7%) CIN II. Of the 80 cervical microbial infections, 62 (77.5%) were yeast cell and 18 (22.5%) *T. vaginalis*. One thirty four (55%) participants had no history of Pap smear screening of which 84 (62.7%) were 20–40 years. Use of IUCDs (OR: 2.47, 95% CI 1.3–4.6) was associated with cervical inflammation.

**Conclusions:**

CIN I was the predominant cervical precancerous change. There is need to scale up cervical screening test to capture all categories of women.

## Background

Cervical cancer is a leading cause of morbidity and mortality among women of the reproductive age especially in low resource limited countries. Widespread screening programs, a catalyst for early detection and management of cervical precancerous changes, significantly reduce cervical cancer incidence [1]. However, these screening programs are limited in Africa and cervical precancerous changes data is scarce [2]. In Kenya, cervical cancer is the leading cancer among women in the reproductive age group [3] nevertheless the screening coverage is at 3.2% [4]. An estimated 4802 women are diagnosed with cervical cancer annually of which 51% (2451) die from the disease [3]. On the other hand, cervical microbial infections have not been quantified.

The recommended cervical cancer screening age is from 25 years and re-screening period is every 5 years except for at risk population [5]. However, cervical screening is mainly conducted in private health facilities which are out of reach to many women in this resource limited setting. We determined the prevalence of cervical precancerous changes and selected cervical microbial infections among women attending FHOK Clinic in Thika for Pap smear testing.

## Methods

### Study design and setting

This was a hospital based cross-sectional study carried out in FHOK Clinic in Thika town. The clinic serves a wide urban and peri-urban population across Kiambu County, Muranga County as well as neighboring outskirts of Nairobi County. The facility is recognized as a centre of excellence in female reproductive health services along with an established quality assured Pap smear screening program.To calculate the sample size, we used a prevalence of 50% estimated by Fisher and Van Belle [6] as no previous study had been done in the area.

### Characteristics of participants

Study participants were consenting women aged 15 years and above seeking Pap smear test at the health facility from November 2013 to January 2014 who had not taken antibiotics within 72 h before study enrollment.

### Data collection

Study participants were interviewed using a structured questionnaire to collect socio-demographic and reproductive health information. This was followed by collection of Pap smear and High Vagina Swab (HVS) samples. Pap smears specimens were processed and stained through the Papanicolaou staining technique [7] for detection of *Candida* infection, *actinomyces* and cervical precancerous changes using Cervical Intraepithelial Neoplasia (CIN) reporting system. High vaginal swabs were processed for wet mount and Gram stain [8] examination for the diagnosis of cervical microbial infections; *Trichomonas vaginalis* infection was indicated by microscopic observation of motile *trichomonads* in wet mount, Gram negative diplococci signified gonorrhea infection and presence of pseudohyphae or hyphae or yeast buds denoted *Candida albicans* infection in Gram stain. Presence of more than 10 white blood cells under microscopic high-power field examination of HVS was classified as cervical inflammation. Both specimens were examined using a light microscope.

### Data analysis

Data generated was entered in Microsoft Excel 2010 and analysed using Epi info version 3.5.1. Univariate analyses were calculated for both the dependent and independent variables. The association between socio-demographic characteristics and stages of cervical precancerous changes and cervical microbial infections were determined using linear regression for age, age at first sex experience and number of births; Chi square for comparing inflammation among the IUCDs users and non-IUCDs users; and fishers exact for marital status, literacy level, employment status, mode of contraception and history of Pap test.

## Results

### Demographics and socio-economics characteristics of participants

A total of 244 participants were interviewed and their Pap smear and HVS specimens examined. Their mean age was 42 (SD: 10.6) years, 134 (55%) had no history of previous Pap smear screening of which 84 (63%) falls within 20–40 years age group; 21–30 age group at 27% (36) and 31–40 age group at 36% (48). The mean age at first sexual experience was 20 (SD: 3) years, 230 (94%) parous of which 22.1% had 2 children, 159 (65.2%) were using various forms of contraceptives with Intrauterine Contraceptive Devices (IUCDs) being used by majority (23.8%) of the study participants (Table 1). Two hundred (81.9%) were married; 188 (94%) in monogamous unions and 12 (6%) in polygamous relationships, 196 (80.3%) were employment, 240 (98.4%) had attained basic formal education; 74 (30.3%) primary education, 84 (34.4%) secondary education and 82 (33.6%) post-secondary education.

**Table 1:** Distribution of Mode of Contraception among women seeking Pap smear screening test, FHOK Clinic in Thika, Kenya, 2014

Mode of contraception	Frequency (%)	Cumulative %
Pills^a,c^	30 (12.3)	12.30
Norplant^a,d^	4 (1.6)	13.90
Lactation amenorrhea (LAM) ^b^	2 (0.8)	14.80
IUCDs^b^	58 (23.8)	38.50
Natural^b^	12 (4.9)	43.40
Tubal Ligation (TL)^b^	24 (9.8)	53.30
None^b^	72 (29.5)	82.80
Condom^b^	8 (3.3)	86.10
Jadelle (JD) ^a,d^	8 (3.3)	89.30
Depo Provera (Depo) ^a,e^	26 (10.7)	100.00
Total	244	100

^a^Hormonal Contraceptives and ^b^Non-Hormonal Contraceptive

^c^Oral Contraceptives, ^d^Implants and ^e^Injectable

### Cervical precancerous changes

Fourteen (5.7%) participants presented with cervical cell abnormalities; 12 (4.9%) with cervical precancerous changes and 2 (0.8%) with viral changes. Of the 12 participants with cervical precancerous changes, 10 (83.3%) had CIN I and 2 (16.7%) had CIN II, with a mean age of 46 (SD: 8) years. Six (50%) had attained post-secondary education level, 10 (83%) were married, mean age at first sexual intercourse was 19 (SD: 2.3) years, 4 (33%) had their first sexual experience <18 years, 10 (83%) had experienced child birth with 8 (67%) having 3–5 children. Ten (83%) had observed the five yearly Pap smear screening recommendation in Kenya [5], that is, had a pap test between 2008 and 2013. Four of the study participants with CIN I, were having their first Pap screening. All (2) women with CIN II had previous screening in the year 2009.

There was no difference in the odds (OR: 1.11, 95% CI: 0.23–5.23) of cervical precancerous changes in women who were married and that of non-married women (single, separated and divorced). No significant association with precancerous changes was found with age, education level, employment status, age at first sexual intercourse, number of births, mode of contraception and history of Pap smear screening on the study participants.

### Cervical inflammation

The prevalence of cervical inflammation was 238 (97.5%) while the prevalence of cervical microbial infection was 80 (32.8%). Of the 80 with cervical microbial infection, 62 (77.5%) were yeast cell and 18 (22.5%) were *T. vaginalis* infections. No actinomyces or gonorrhoea infection was identified. The distribution of yeast cell and T. vaginalis infections among study participants was statistically significant (*P* < 0.05). Among the different techniques employed in this study, HVS wet preparation had the highest positivity rate to inflammatory changes and their causes (Table 2). Of the 58 women using Intrauterine Contraceptive Devices (IUCDs), 26 (45%) exhibited cervical inflammation in their Pap smear results. Use of IUCDs (OR: 2.47, 95% CI 1.3–4.6, comparator were non-IUCDs users) was associated with having cervical inflammation.

**Table 2:** Comparison of different techniques (Pap smear, Gram stain and HVS) used for diagnosis of cervical inflammation and their causes among women seeking Pap smear screening test, FHOK Clinic in Thika, Kenya, 2014

Micro-organism / cells observed		Diagnostic test used	
	Pap smear	HVS^a^ Gram stained	HVS Wet preparation
*Candida ablican* /yeast cells	6	60	62
*T. vaginalis*	0	0	18
Gram negative intracellular diplococcus	0	0	0
Actinomyces	0	0	0
Pus cells/ Polymophs	72	0	238

Legend: ^a^High Vagina Swab

## Discussion

We observed two levels of cervical precancerous changes; CIN I and CIN II. Cervical intraepithelial neoplasia I (mild dysplasia) was the major dysplasia (83%) observed among the study participants during the study period. *Candida* and *T. vaginalis* were identified as cause of cervical inflammation among the study participants.

This study reported a low prevalence of cervical cell abnormalities (4.9%); 83.3% (10) Low-grade cervical lesions and 16.7% (2) high-grade cervical lesions. The results are comparable to a study done at an outpatient reproductive health clinic in Pakistan whereby 4.6% (32) cases had dysplastic changes of which 56.25% (18) were LSIL and 43.75% (14) HSIL [9]. Similar frequency of dysplastic smears were reported by Inamullah et al. [10]; 5% dysplasia of which 75% (6) were LSIL and 25% (2) HSIL, in a study done in Pakistan among women presenting with chronic discharge. Additionally, a study in women seeking care at a reproductive health clinic in Pakistan from February 2009 to February 2010, revealed dysplasia frequency of 3.7% (10 cases) of which 80% (8) had LSIL while 20% (2) HSIL [11]. These precancerous changes were detected among women on routine screening within age 34–60 years. This is important in Kenya; a country where 60% of cancer victims are below 70 years and 70–80% of the diagnose happening in late stages owing to lack of awareness, inadequate diagnostic facilities, lack of treatment facilities, inadequate personnel to manage invasive stages, high cost of treatment and high poverty Index [5, 12].

More than half (55%) of the study participant were having their first ever Pap smear screening of which 36% were 31–40 years. This falls within the reproductive age where most of the screening interventions should be done. The Centers for Disease Control and Prevention (CDC) epidemiological study found that 78% of the cervical cancer cases were diagnosed in women 30–39 years [13]. It is therefore not surprising that in Africa, where screening rates are low or non-existent, majority of women present with advanced disease. A study done by Sheikh and Manhua [14] in Saudi Arabia from January 2009 to January 2011 reported 83% (1224) of study participants had never been screened with Pap smear which was higher compared to our findings. In the current study, low uptake of Pap smear screening may be due to lack of awareness and/or shying away due to the invasive nature of Pap smear sample collection procedure. This illustrates that Pap smear screening programs need to be scaled up to reach more women and attain higher screening rates.

According to Burkadze and Gulisa [15], three out of four women who develop cervical cancer each year have never had a Pap smear or had not had one within the recommended intervals. Four cases (2.98%) of CIN I in this study had never had a Pap smear test before although not having a previous Pap smear test was not significantly associated with cervical precancerous changes in our study. Sheikh and Manhua [14] reported similar rates of cervical abnormalities among participants who were having Pap smear screening for the first time in Saudi Arabia, 2.91% (43). This suggests that earlier Pap smears screening would have meant earlier detection leading to an increased rate of recession and hence low mortality. Countries, such as Taiwan, with widespread organized screening programmes for cervical cancer, have substantially reduced cervical cancer incidence and mortality by 60–90% as well as increased survival rates [16–18]. Unfortunately in resource-limited countries, where Pap screening has not been effectively implemented, cervical cancer remains a major public health problem [19].

High prevalence of inflammatory changes (97.5%) was reported in this study. Cervical microbial infections accounted for 32.8% of the inflammation cases. These results contradict previous findings by a study at National Institute of Health, Islamabad in Pakistan where lower prevalence of inflammatory changes (55.31%) was reported [20]. Similarly, Rubia and Huma [11] found 55.7% (156) frequency of inflammation and 55% (88) was documented in another study by Inamullah et al. [10]. This higher prevalence of inflammatory changes among women with high literacy level and majority working women could be due to improved health seeking behaviour due to women empowerment.

Vaginal infections are common cause of inflammation of the genital tract in all women; some are associated with sexual activity while others, such as vaginal candidiasis, are not. In this study, Candida (25.4%) and Trichomonas vaginalis (7.4%) were the specific causes of inflammation identified in the High Vaginal Swab wet preparation. Differing findings have been documented in other studies. Bhojani and Garg [21] reported 0.5% prevalence of both Candida and Trichomonas vaginalis while Claeys et al. [22] reported a prevalence of 19.1% for Candida and 10.1% for Trichomonas vaginalis. Candida was the predominant (25.4%) specific cause of inflammation. This could be due to the fact that vaginal Candida species are emerging as significant opportunistic organisms that have increased over the past few decades attributed to both inappropriate use of antibiotics, increased use of hormonal contraceptives and the increasing population of immune-compromised individuals.

Detection of *Trichomonas vaginalis*, a sexually transmitted micro-organism, was suggestive of existence of potential for HIV transmission and the probable explanation would be due to low utilization of barrier method of contraception such as condom, which would provide protection from sexually transmitted diseases as well as cervical cancer. Actinomyces infection is usually detected in IUCD users. The infection was not reported in even among the IUCD users. This can be attributed to the fact that all IUCD users had a history of a previous Pap tests and therefore might have been treated previously. However, inflammation characterized by presence of polymorphonuclear cells (granulocytes) was evident in 26 (44.8%) of the IUCD users through Pap smear examination and with a significant statistical association (OR 2.47 at 95% C.I.). Studies in other parts of the world revealed similar association between vaginitis, and use of IUCDs. Sieber and Dietz [23] and Amsel et al., [24] found a strong association of nonspecific vaginitis with the use of IUCDs. This could be related to the insertion method and technique leading to introduction of bacteria into the uterine cavity or the IUCDs mobilizing polymorphonuclear leucocytes since it is a foreign body. Women using IUCDs requires a regular follow up and clinical examination.

## Conclusions

Low prevalence of cervical precancerous changes and high prevalence of cervical microbial infection was reported. Majority of the participants were having Pap smear screening for the first time with the most un-screened persons falling within 21–40 years. Yeast cell was the predominant microbial organism detected with most causes of inflammation remaining non-specific.

### Recommendation

There is need to scale up cervical cancer screening awareness in order to capture all categories of women in all age groups in the cervical cancer screening programs. Pap smear screening and high vaginal swab should be offered as one package to identify both precancerous changes and microbial infections not as stand-alone tests. Further studies on the non-specific cause of cervical inflammation should be conducted.

## Abbreviations

CI: Confidence interval
CIN: Cervical Intraepithelial Neoplasia
FHOK: Family Health Option Kenya
HIV: Human Immunodeficiency Virus
HSIL: High grade squamous intraepithelial lesion
HVS: High Vagina Swab
IUCDs: Intrauterine Contraceptive Devices
KUERC: Kenyatta University Ethics Review Committee
LSIL: Low grade squamous intraepithelial lesion
NGPMCBPC: National Guidelines for Prevention and Management of Cervical, Breast and Prostate Cancer
OR: Odds Ratio
Pap smears: Papanicolaou smear
SD: Standard deviation
T. vaginalis: Trichomonas vaginalis
